# Impulsivity in Parkinson’s disease patients treated with subthalamic nucleus deep brain stimulation—An exploratory study

**DOI:** 10.1371/journal.pone.0248568

**Published:** 2021-03-12

**Authors:** U. Pham, I. M. Skogseid, A. H. Pripp, E. Bøen, M. Toft

**Affiliations:** 1 Division of Mental Health and Addiction, Psychosomatic and CL Psychiatry, Oslo University Hospital, Oslo, Norway; 2 Department of Neurology, Oslo University Hospital, Oslo, Norway; 3 Faculty of Health Sciences, Oslo Metropolitan University, Oslo, Norway; 4 Department of Biostatistics, Epidemiology and Health Economics, Oslo University Hospital, Oslo, Norway; 5 Faculty of Medicine, University of Oslo, Oslo, Norway; Sapienza University of Rome, ITALY

## Abstract

**Background:**

Deep brain stimulation of the subthalamic nucleus (STN-DBS) is a recognized treatment in Parkinson’s disease (PD). Knowledge is still limited regarding the possible impact of STN-DBS on personality traits and the personality characteristics of PD patients who undergo surgery.

**Methods:**

To assess personality traits in relation to STN-DBS we did an ancillary protocol as part of a prospective randomized study that compared two surgical strategies. Patients were assessed with the Temperament and Character Inventory (TCI), the Urgency, Premeditation, Perseverance and Sensation Seeking impulse behavior scale, the Eysenck Personality Questionnaire (EPQ) and the Toronto Alexithymia Scale preoperatively and after one year of STN-DBS. EPQ and TCI baseline scores were compared with mean scores of healthy reference populations.

**Results:**

After 12-months of STN-DBS, there was a significant decline in Persistence compared to baseline. Preoperatively, the STN-DBS patients had significantly lower Persistence and Self-Transcendence scores, and significantly higher scores on Novelty-Seeking, Self-Directedness, Cooperativeness and on Social Conformity than referenced populations. No difference was found in Neuroticism or Harm-Avoidance scores. The baseline prevalence of alexithymia was low and at 1-year follow-up there was no significant change in alexithymia scores.

**Conclusions:**

We found a higher baseline level of impulsivity in PD patients who underwent STN-DBS. After one year of STN-DBS, our results indicated that the treatment may affect the patients’ personality by increasing certain aspects of impulsivity. There was no effect on alexithymia. The preoperative personality profile of PD patients might influence the outcome of STN-DBS.

## 1. Introduction

Parkinson’s disease (PD) is a neurodegenerative disorder characterized by motor and non-motor symptoms. Deep brain stimulation (DBS) of the subthalamic nucleus (STN-DBS) is an effective treatment option for motor symptoms in PD. Patients eligible for STN-DBS are clinically in an advanced disease stage with motor fluctuations, dyskinesias, and/or marked refractory tremor [1]. Prior research on cognitive and behavioral aspects of PD has shown that various neuropsychiatric symptoms may arise, worsen or improve after this intervention [2]. The relationship between personality and DBS treatment is not fully investigated and knowledge about the impact STN-DBS might have on PD patients’ personality is still limited. While a consensus has been established regarding the neurological criteria of PD patients eligible for STN-DBS [3], the personality characteristics of PD patients undergoing STN-DBS have not been sufficiently explored. To our knowledge, no previous study has conducted comprehensive investigation on personality characteristics in PD patients undergoing STN-DBS.

In earlier literature, patients with PD have often been linked to a specific personality type, sometimes referred to as the “Parkinsonian personality”. Personality traits commonly used to characterize PD patients are mental rigidity, introversion, and cautiousness [4]. Studies that applied the Cloninger’s Temperament and Character Inventory (TCI) [5] and Tridimensional Personality Questionnaire (TPQ) [6] have shown lower Novelty-Seeking scores and higher Harm-Avoidance scores in PD patients compared to controls [4,7,8]. Research that assessed the personality traits of PD patients using the Big-Five inventory suggested that the subjects present higher levels of Neuroticism and lower levels of Openness and Extraversion than controls [4,9]. Another personality aspect investigated in PD is alexithymia, which refers to difficulties with identifying and describing feelings, and an externally oriented way of thinking. Recent studies have suggested a higher prevalence of alexithymia in PD patients than in the general population [10].

Previous studies that used the TCI to investigate the possible effects of STN-DBS on personality have shown diverse results. One study did not find any modification of personality traits after 24 months of STN-DBS [11], whereas another found some modest alteration only on the subdimension level of Novelty-Seeking after 6 months of DBS [12]. More recently, Lhommée and co-workers found an increase in Harm-Avoidance and a decline at the subdimension level on Novelty-Seeking and Reward-Dependence after one-year of DBS [13]. In a mixed study design, with both qualitative interviews and quantitative measurements, Lewis and colleagues found that personality changes were subjectively perceived by six of 27 patients and by 10 of 23 caregivers. The reported personality changes were, however, not sufficiently reflected in the quantitative measurements used in the study [14].

In our previous work, we showed that STN-DBS in PD patients was associated with personality changes in the direction of increased impulsivity three months after surgery [15]. We found a significant decline on the TCI [5] dimensions Persistence and Self-Transcendence, and a significant increase in the informant ratings on the dimension (Lack of) Premeditation of the Impulse Behavior Scale; Urgency, Premeditation, Perseverance and Sensation Seeking (UPPS) [16].

In this follow-up study, we wanted to investigate the impact of STN-DBS on PD patients’ personality and to investigate whether the changes in personality traits that we previously found after three months of STN-DBS [15] were sustained at one year. Further, our goal was to explore the baseline personality characteristics and investigate the frequency of alexithymia in PD patients undergoing STN-DBS.

## 2. Methods

### 2.1. Subjects

Initially 60 patients were included in a prospective randomized study that compared two surgical strategies. Three patients had surgical site infections with discontinuation of neurostimulation due to hardware removal before the 3-months follow-up. Two patients were lost to the 1-year follow-up. Consequently 55 patients completed the study protocols at 1-year follow-up. To assess personality traits in relation to STN-DBS we did an ancillary protocol. Detailed study description of the design and the main results on primary endpoints have been published previously [17], and will therefore only be described briefly here. The inclusion and exclusion criteria and baseline characteristics are shown in Table 1.

**Table 1:** Criteria for study participation and baseline characteristics of study population.

**Inclusion Criteria**:	
Parkinson’s disease according to the UK Brain Bank criteria	
Age < 75 years	
Disease duration ≥ 5 years	
MDS-UPDRS motor score ≥ 20 points in the medication-off state	
> 30% reduction of non-tremor motor score in medication-on state (range 0 to 108) or severe levodopa unresponsive tremor	
Marked motor fluctuations with or without troublesome dyskinesias, and/or severe tremor, and/or intolerable side effects of dopaminergic drugs	
Failure of best oral medical treatment to sufficiently control symptoms	
Mattis Dementia Rating Scale score >130 (maximum 144)	
**Exclusion Criteria**:	
Previous surgery for Parkinson’s disease	
Marked axial motor symptoms unresponsive to levodopa	
Brain MRI showing marked atrophy or white matter changes	
Increased risk of bleeding	
Comorbidities with short life expectancy Other surgical contra-indications	
Dementia	
Severe untreated psychiatric disorders (including psychosis, major depression or hypomania / mania)	
Insufficient understanding of the Norwegian language (preventing participation in the psychiatric and neuropsychological evaluations)	
**Baseline Characteristics of the Study Population**	
Female (n)	15
Male (n)	45
Age in Years (mean, range)	62 (44 to 71)
Disease duration in years (mean, range)	11 (4 to 23)
Mattis Dementia Rating Scale	142 (131 to 144)
Levodopa-equivalent dosage in mg (LEDD)	1291 (428 to 2490)
MDS UPDRS III-OFF (mean, range)	47 (23 to 78)
MDS UPDRS III-ON (mean, range)	13 (1 to 45)
Hoehn & Yahr-OFF (median, range)	3 (2 to 5)
Hoehn & Yahr-ON (median, range)	2 (1 to 3)

### 2.2 Neurological evaluations

The Movement Disorder Society revision of the Unified Parkinson’s Disease Rating Scale (MDS-UPDRS I-IV) was used to investigate motor symptoms (III), motor complications (IV), and non-motor and motor experiences of daily living [18]. MDS-UPDRS III was scored after an overnight withdrawal of dopaminergic drugs (medication-off), and after a levodopa dose approximately 1.5 times the patient’s usual morning dose (medication-on).

### 2.3 Personality trait assessments

To assess personality traits, we applied the 125-items version of the TCI (TCI-125) [2], the UPPS [16], The Neuroticism and the Lie subscale of the Eysenck Personality Questionnaire (EPQ-N and EPQ-L) [19] and the 20-items version of the Toronto Alexithymia Scale (TAS-20) [20].

TCI-125 [2] measures seven personality dimensions, including four dimensions of temperaments: Novelty-Seeking, Harm-Avoidance, Reward-Dependence and Persistence, and three dimensions of characters: Self-Directedness, Cooperativeness and Self-Transcendence. Higher scores indicate a greater tendency for that particular trait. Novelty-Seeking describes exploratory activity, impulsiveness, extravagance, disorderliness. Harm-Avoidance characterize excessive worrying, pessimism, fearfulness. Reward-Dependence defines the response to signals of reward; social approval, and social support. Persistence refers to perseverance. Self-Directedness refers to self-determination, adaptation and regulation of behavior. Cooperativeness describes agreeableness in relationships. Self-Transcendence involves the expansion of personal boundaries and spirituality. The normative sample used for comparison was based on the TCI scores from a large Italian cross-sectional study including 1430 healthy individuals [21].

The UPPS [16] is a 45 items inventory measuring four dimensions of impulsivity; Urgency, Premeditation (lack of), Perseverance (lack of), and Sensation Seeking. Higher scores indicate higher levels of impulsivity. Urgency refers to the tendency to experience strong impulses. Premeditation (lack of), refers to the tendency to think and reflect on the consequences. Perseverance (lack of), refers to the ability to remain focused on a task. Sensation Seeking, incorporates a tendency to enjoy and pursue exiting activities. The informant version of the UPPS was also administered.

The EPQ-N and EPQ-L [19] contains 44 items, with yes or no to assess the two dimensions of personality, neuroticism (N) and social conformity (L). Neuroticism refers to emotional instability, worry and tenseness. People who score high on EPQ-N are anxious, depressed and react strongly to aversive stimuli, whereas people with low neuroticism scorers are considered stable and relatively unreactive. Higher EPQ-L scores reflect a tendency toward social conformity and social desirability. A high negative correlation (r > -0.5) between high EPQ-L and low EPQ-N can be interpreted as dissimulation or a tendency to underreport psychosocial problems. The normative sample used for comparison of the EPQ results consisted of 802 healthy controls representative of the healthy adult population in Norway [22].

The TAS-20 [20] was administrated to assess alexithymia. The total alexithymia score is the sum-score of difficulty in identifying feelings (DIF), difficulty in describing feelings (DEF), and external oriented thinking (EOT). Established cut-offs are: 51 = non-alexithymia, scores between 52 to 60 = possible alexithymia, and > 61 = alexithymia [20].

### 2.4 Depression and anxiety

Anxiety and depressive symptoms were assessed by the Hospital Anxiety and Depression Scale (The HADS-A and HADS-D) [23]. Recommended cut-off scores for both scales are 8–10 for doubtful cases and > 10 for definite cases [23].

### 2.5 Statistical analysis

The changes of scores from baseline to 12-months follow-up were studied using paired sample Student’s *t*-tests. Cohen’s *d* for paired sample t-test was calculated for estimation of effect size. A Cohen’s *d* of >0.2 is considered a small effect, >0.5 a medium effect and >0.8 a large effect. We compared our sample with the normative samples studied by Eysenck et al. [22] and Delvecchio et al [21] using a t-test based on number of participants and the estimated mean and standard deviation in each sample.

The covariation between scores was analyzed with Pearson’s correlation coefficients. The significance level was defined as p-value less than 0.05. Many variables were explored in this study. Due to the explorative nature of the study, we did not make adjustments for multiple comparisons. However, we were aware of the increased risk of type I error due to many comparisons and analyzed results critically.

The TCI-125 was completed by 47 patients both preoperatively and at 12 months follow-up. The baseline scores were compared to mean scores of 262 healthy controls in the age group 50–69 years in the TCI-125 population study [21]. For the UPPS, 32 self-reported protocols and 17 informant protocols were valid both preoperatively and postoperatively. On the EPQ, 12 patients had more than 20% missing scores, either at baseline or at 12-month follow-up, leaving 43 valid protocols for pairwise comparison. Preoperatively, 12 of 15 women and 35 of 45 men had completed the EPQ. The baseline values were compared with the EPQ mean scores of 425 females and 377 males in the reference norm population [22]. 43 patients had completed the TAS and 48 patients had completed the HADS, both preoperatively and at 12 months follow-up.

### 2.6 Ethical aspects

The study was conducted according to the declaration of Helsinki. Informed written consent was obtained from all participants. The study was approved by the Regional Committee for Medical and Health Research Ethics (REC South East, project no. S-00944c 2009/805).

## 3. Results

### 3.1. Neurological evaluations

The MDS-UPDRS III-medication OFF scores improved from mean (SD) 49 [13] preoperatively to 20 [9] at one-year follow-up, and LEDD was reduced from 1301 (441) preoperatively to 639 (328) at one-year follow-up (both *p*< 0.001).

### 3.2 Personality trait assessments

There were no differences in the somatic adverse events or neuropsychiatric adverse events between the two randomized group from the parent study at the different time points [17]. Thus, for the purpose of this paper we analyzed the results of the personality assessments from the two groups together.

After one year of STN-DBS, we found a significant decline in the temperament score Persistence compared to baseline (Table 2). No significant correlation was found between this decline in Persistence score and the improvement of MDS-UPDRS III-OFF (Pearson correlation coefficient, r = -0.07, p = 0.6), MDS-UPDRS III-ON (r = 0.1, p = 0.5), or the reduction of dopaminergic medication (r = -0.2, p = 0.16).

**Table 2:** The Temperament and Character Inventory (TCI-125) scores of normative sample, preoperative scores and at 12 months follow-up in our study population.

TCI dimensions	Normative sample	Preoperative Mean ± SD	12-month follow-up Mean ± SD	Mean diff. ± SD preop.– 12 months	Cohen’s d	P-value (preoperativ-12-month)
Novelty Seeking	8.3 ± 3.2	9.3 ± 3.3	9.8 ± 3.0	0.5 ± 2.9		0.27
Harm Avoidance	9.1± 4.6	8.4 ± 3.9	9.0 ± 3.7	0.6 ± 3.0		0.15
Reward Dependence	8.4 ± 2.9	9.2 ± 2.7	8.6 ± 2.3	-0.6 ± 2.3		0.11
Persistence	3.1 ± 1.5	2.4 ± 1.6	1.8 ± 1.5	-0.6 ± 1.3	0.5	< 0.01
Self-Directedness	17.9 ± 5.1	20.8 ± 2.9	20.0 ± 3.8	-0.8 ± 3.0		0.08
Cooperativeness	19.0 ± 3.5	21.0 ± 3.2	20.4 ± 2.8	-07 ± 2.6		0.08
Self-Transcendence	6.3 ± 3.6	3.8 ± 3.1	3.4 ± 3.0	-0.3 ±2.0		0.24

At baseline the PD patients in this study had significantly lower mean scores on Persistence (p = 0.003) and Self-Transcendence (p < 0.001) and significantly higher scores on Novelty-Seeking (p = 0.04), Self-Directedness (p < 0.001), and Cooperativeness (p < 0.001) compared to the mean scores of the TCI-125 reference population [21]. There was no significant difference in the dimensions Harm-Avoidance and Reward Dependence between the PD patients and the reference population.

One-year after STN-DBS there was no significant change in the self-report scores of the impulsivity facets of the UPPS. The informant reports showed a trend towards increase in the facet (lack of) Premeditation, with a p-value of 0.09 (Table 3).

**Table 3:** The Urgency, Premeditation, Perseverance and Sensation Seeking impulse behavior scale (UPPS) self-reported and informant scores preoperatively and at 12 months follow-up.

UPPS subscales	Preoperative Mean ± SD	12-months follow-up Mean ± SD	Mean diff. ± SD preop.– 12-months follow-up	p-value (preop.– 12 months)
**Urgency**				
Self-report	21.8 ± 6.0	20.8 ± 6.2	- 1.0 ± 4.7	0.25
Informant	20.4 ± 6.1	21.8 ± 8.3	1.4 ± 6.0	0.36
**(Lack of) Premeditation**				
Self-report	22.6 ± 6.0	23.6 ± 3.8	1.0 ± 6.4	0.38
Informant	21.3 ± 6.0	23.2 ± 7.3	1.9 ± 4.5	0.09
**(Lack of) Perseverance**				
Self-report	19.2 ± 4.5	19.7 ± 4.5	0.5 ± 4.6	0.56
Informant	19.3 ± 5.7	20.9 ± 7.0	1.6 ± 6.1	0.14
**Sensation-Seeking**				
Self-report	22.4 ± 7.9	23.7 ± 9.2	1.3 ± 5.3	0.19
Informant	20.2 ± 6.6	18.2 ± 3.9	2.0 ± 5.3	0.29

At one-year follow-up, neither the EPQ-N nor the EPQ-L had changed significantly compared to baseline (Table 4). There was a low and non-significant negative correlation between EPQ-L and EPQ-N (preoperatively; r = -0.1, p = 0.5, 12- month follow-up; r = -0,2, p = 0.08).

**Table 4:** Eysenck Personality Questionnaire (EPQ) scores of normative sample, and scores from preoperative and 12 months follow-up in our study population.

EPQ subscores	Norm women Mean ± SD	Preoperative women Mean ±SD	Norm men Mean ±SD	Preoperative men Mean ± SD	Preoperative total Mean ± SD	12-months follow-up Mean ± SD	Mean diff. ± SD preop.– 12 months	p-value (preop. –12 months)
EPQ-Neuroticism	8.2 ± 4.3	5.9 ± 2.8	6.2 ± 4.5	5.6 ± 3.4	5.7 ± 3.3	5.6 ± 4.3	- 0.1 ± 3.1	0.8
EPQ-Lie	8.1 ± 3.8	10.3 ± 3.7	6.7 ± 3.9	9.7 ± 3.8	9.6 ± 3.6	10.4 ± 4.4	0.7 ± 2.7	0.1

The preoperative EPQ-L scores of both genders were significantly higher than the reference population for both women and men (p = 0.04 and p < 0.001, respectively), whereas for the preoperative EPQ-N scores no significant difference compared to the general population was found (women: p = 0.06; men: p = 0.43) [22].

According to established cutoff scores of the TAS, the PD patients in this study had mean scores below the cutoff for alexithymia both preoperatively and at one-year follow-up. Preoperatively 7.8% of patients had scores above cutoff (> 61). From baseline to one year of STN-DBS there was no significant change on either the TAS total score or on the subscales DIF, DEF or EOT (Table 5).

**Table 5:** Toronto Alexithymia Scale (TAS-20) total score and subscores.

	Preoperative Mean ± SD	12- months follow-up Mean ± SD	Mean diff. ± SD preop.– 12 months	p-value (preop.– 12 months)
Total TAS-20	44.9 ± 10.2	45.9 ±10.9	1.0 ± 1.3	0.44
DIF	13.1 ± 4.8	13.3 ± 4.5	0.2 ± 4.3	0.78
DEF	12.2 ± 3.9	12.0 ± 4.41	0.2 ± 3.4	0.69
EOT	19.6 ± 5.3	20.6 ± 5.1	1.0 ± 4.1	0.10

DIF—Difficulty in identifying feelings, DEF—Difficulty in describing feelings, EOT—External oriented thinking. SD—standard deviation.

### 3.3. HADS Depression and anxiety scores

The mean HADS-Depression score increased from 3.3 ± 2.8 at baseline to 4.2 ± 3.3 at the 12 months follow-up (p = 0.003, Cohen’s effect size = 0.3), whereas the mean HADS-Anxiety was 3.7 ± 2.8 preoperatively and 3.0 ± 3.3 at 12 months (p = 0.16). Although the mean HADS-Depression score increased significantly after one year of STN-DBS, the mean score for both HADS-Depression and HADS-Anxiety scores was below established cutoffs for depression and anxiety both preoperatively and at the 12 months follow-up.

## 4. Discussion

In this follow-up study, we have investigated the impact of STN-DBS on PD patients’ personality and studied whether the changes in personality traits that we previously found after three months of STN-DBS [15] sustained at 1-year follow-up. Another aim was to evaluate the personality characteristics of PD patients undergoing STN-DBS.

We also wanted to determine the frequency of alexithymia in the study population.

After one year of STN-DBS the TCI-125 findings were consistent with our previous findings [15], and showed a significant decline in the mean score of the character dimension Persistence compared to baseline. We did not find any correlation between this decline in Persistence and the reduction of the dopaminergic medication or any of the motor outcome scores. Persistence is a person’s ability to persevere and keep trying, even when things get difficult. Low Persistence is an adaptive strategy when reward contingencies change rapidly and may be maladaptive when rewards are infrequent but occur in the long run [24]. Individuals who are low in Persistence are described as changeable, irresolute, and easily discouraged [5] especially when rewards are intermittent. Similar features have been described in PD patients with Impulse Control Disorders (ICDs) which includes Gambling, Compulsive shopping, Compulsive sexual behavior and Binge eating [25]. Common characteristics of the cognitive dysfunction in PD patients with ICDs involve abnormalities related to cognitive flexibility, reversal learning and reinforcement. PD patients with ICDs exhibit a preference for more immediate rewards and a tendency to undervalue delayed rewards [26]. A cross-sectional study [25] found higher Novelty-Seeking and impulsivity in ICD patients. Persistence and other personality traits of the TCI were however not examined in that study. To our knowledge the relationship between a comprehensive assessment of personality traits and ICDs is still unexplored in the PD population.

In the study of Lhommée et al. [13], the authors suggested that the observed significant changes in personality traits may be related to postoperative tapering of dopaminergic treatment. However, despite significant reduction of the dopaminergic medication after one year of STN-DBS, we did not find any decline in the Novelty-Seeking scores. Also, the Harm-Avoidance dimension in our study remained unchanged, unlike the findings of Lhommée [13].

We have previously reported a significant increase in the informant report of the UPPS (lack of) Premeditation after three months of STN-DBS [15]. After one year of STN-DBS, the patient’s close relatives still reported higher scores on (lack of) Premeditation which indicated that the informants had observed the patient’s tendency to impulsive behavior. However, the findings at one year did not reach statistical significance. The lack of significant changes in the informant ratings at one-year follow-up might raise the question whether the increased impulsivity reported at three months were transient phenomena or might have been due to temporary hypomanic reactions induced by STN-DBS. Previous studies have described adverse mood effects related to DBS. Behavioral changes such as hyperactivity, disinhibition and impulsivity have been reported to occur in particular immediately after surgery [2]. A more likely explanation may be that the patients’ increased impulsive behavior may have been adapted as “ego-syntonic” and no longer noticed by family members. However, these psychological mechanisms are complex, and difficult to measure. Clinicians and researches in the field of bipolar disorders struggle with similar challenges when trying to identify and capture the phenomenon of subthreshold hypomanic features [27].

Regarding the preoperative personality characteristics of the PD patients who underwent STN-DBS, the TCI-125 baseline profile showed significantly lower Persistence and Self-Transcendence scores, and significantly higher scores on Novelty-Seeking, Self-Directedness and Cooperativeness than comparative norms. Lower Persistence and higher Novelty Seeking scores indicate an elevated level of impulsivity at baseline. The preoperative hyperdopaminergic state of the PD patients might be associated with the increased impulsivity [28].

The baseline prevalence of alexithymia in our study population was low (7.8%), and after one year of STN-DBS there were no significant change on either the TAS total score or on the subscales DIF, DEF or EOT. Previous studies have found a high frequency of alexithymia, both in general PD populations (21.4%) [29] and at baseline in PD patients who underwent STN-DBS (17.9% -31.6%) [30,31]. Regarding the potential impact of STN-DBS on alexithymia, previous studies have shown divergent result. Dafsari et al. [30] found a decrease of alexithymia at 5 months follow-up, while the results of Castelli et al. did not suggest any effect of STN-DBS on alexithymia [31].

Our findings have not confirmed previous research stating that PD patients are more neurotic than healthy controls [9]. The lower EPQ-N scores found in our PD patients imply emotional stability [19]. This might be explained by their lower level of depression and anxiety. The significantly higher EPQ-L found in the STN-DBS patients may indicate the tendency to present oneself as social desirable with a high moral standard [19]. This finding might to some degree reflect the traditional description of the “Parkinsonian personality”, where PD patients have been characterized as moralistic and social conform [32]. Neuroscientific studies have suggested that the neural basis of conformity in trusting behavior are similar to the mechanisms implicated in reward learning [33]. These mechanisms might provide an explanation for the high social conformity scores found in the PD patients. Higher scores of the EPQ-L could also be interpreted as “fake good”. There is considerable empirical evidence to indicate that individuals who have a high motivation to dissimulate, inflate their EPQ-L scores and suppress their EPQ-N scores, which would lead to a high negative correlation between EPQ-L and EPQ-N scores [34]. PD patients who are evaluated for STN-DBS might have a motivation to “fake good” in order to meet the selection criteria. However, our results did not show any indication of dissimulation.

The divergence between the personality characteristics of the STN-DBS treated PD patients in this study and the personality traits described in more typical (non-operated) PD populations in previous studies [7–10,35], may be due to selection bias. Before being accepted as suitable candidates for STN-DBS, our PD patients had received multidisciplinary evaluation and had been screened both physically, cognitively and mentally. Furthermore, the PD patients selected for STN-DBS in this study differed from a general PD population in several aspects. They were relatively younger (mean age 61) but had reached an advanced disease stage with medically intractable PD and were at the time of operation on average treated with high doses of dopaminergic medication. Most patients had developed motor fluctuations, including dyskinesias. At the same time, they were non-demented (MDRS score >130) and reasonably well-adjusted mentally with no major psychiatric disorders.

With 75% men in this cohort, there might also be a gender bias. Previous research has suggested that there are some differences in certain personality traits between the genders [36]. Studies reviewing characteristics of DBS patients have also found a predomination of men, reporting as potential causes that men were more likely to be referred to surgery and pursue DBS intervention than women [37]. Another explanation may be that people with personality profiles characterized by low Novelty-Seeking, high Neuroticism and high Harm-Avoidance, are less likely to prefer an invasive treatment option like DBS surgery. Previous research has suggested that patients with high Neuroticism are more passive in their health care and treatment preferences than others [38].

## 5. Strengths and limitations

The key strength of this work lies in the comprehensive investigation of personality traits in PD patients before and after one year of STN-DBS, using multiple personality instruments that included self-report inventory as well as complementary information from relatives.

The present study has some limitations that are common for prospective longitudinal studies.

One of the limitations is the absence of a control group of PD patients not treated with STN-DBS. Without a “treatment as usual” control group we cannot rule out the possibility that patients who had been treated with dopaminergic medications alone would have shown the same changes in impulsivity as our STN-DBS group. Although we find it less likely, we cannot dismiss the fact that modifications in personality traits may emerge from progression of the disease itself, rather than the effect of STN-DBS. To obtain a control group of PD patients who matched our group of STN-DBS treated patients at a single DBS center was, however, ethically challenging. One of the main reasons was that eligible PD patients would themselves be candidates for surgery. A further limitation is that we did not attempt to correct for multiple comparisons. This implies that we accept a higher probability of type I errors. The findings should be interpreted with caution since the p-values are based on exploratory analyses.

One of the challenges faced in this study was the failure by participants to complete the self-rated questionnaire at different points in time. The missing values were excluded from the statistical analysis, which reduced the sample size and study power. However, in the patient group with advanced PD, the lack of completion of this very comprehensive survey by some members is to be expected. Although the sample size is limited, it is comparable to the sample size in previous studies about the effects of STN-DBS in PD patients [11,12,30,31].

In this study we have chosen to compare the preoperative TCI-125 scores and the EPQ values of our STN-DBS treated PD patients with normative data from large population studies. Comparable data of TCI-125 and EPQ mean scores from a general PD population are not available. Although we are aware of possible socio-cultural influence on personality, we believe that the Italian TCI- 125 normative data are representative and comparable to our main findings. The TCI have been used extensively in research studies worldwide during the past two decades and have been translated and validated in various countries [39]. In 2006 Miettunen et al. compared the temperament dimensions of the TCI across 20 countries. Their main results indicated that there were few major differences in the mean scores between different countries, and the authors supported the cross cultural use of the TCI [39].

Being a complex concept, personality traits are challenging to assess and quantify. The divergent findings between our study and previous research might be due to differences in methodological procedures and the variation in the personality inventories applied. Taking into consideration the abovementioned factors, our study has its limitations, which means that the study findings should be interpreted with caution.

Overall, the biological mechanisms of the neuropsychiatric effects of STN-DBS in PD are multifactorial, including the role of the STN in both motor and non-motor networks, accuracy of electrode placement, current diffusion, changes in the dopamine replacement therapy and the microlesion effects of the surgery [2,40].

## 6. Conclusions

After one year of STN-DBS, we found a decline of the dimension Persistence compared to preoperatively, despite of a major reduction of dopaminergic medication. These findings implicate that STN-DBS may affect PD patients’ personality by increasing certain aspects of impulsivity.

Preoperatively, the PD patients had significantly higher scores on the TCI dimension Novelty-Seeking and lower Persistence scores compared to healthy controls, which indicate a higher baseline level of impulsivity in the PD patients who have decided to undergo surgery. The PD patients who underwent STN-DBS in this study also seemed to differ from the general PD population in other personality aspects. Our findings did not confirm previous research stating that PD patients are more neurotic, harm-avoidant or have more alexithymia than healthy controls [4,7–10]. However, we found significantly higher EPQ-L scores in the STN-DBS patients group compared to a reference norm population indicating that this group of PD patients have a tendency to present themselves as social desirable with a high moral standard [19]. This might to some degree reflect the traditional description of the “Parkinsonian personality”, where PD patients have been characterized as moralistic and social conform [32].

Further research is needed to obtain more evidence on the impact of STN-DBS on PD patients’ personality. It would be of particular interest to explore the personality characteristics of the PD patients who decide to undergo surgery, and which impact the preoperative personality profile might have for the outcome of STN-DBS, for instance with regard to impulsivity. Such findings could have implications for the future selection of STN-DBS candidates and the clinical follow up care of PD patients who undergoes STN-DBS.
